# New onset type 2 diabetes mellitus risks with integrase strand transfer inhibitors-based regimens: A systematic review and meta-analysis

**DOI:** 10.1016/j.metop.2023.100235

**Published:** 2023-02-14

**Authors:** Violet Dismas Kajogoo, Wondwossen Amogne, Girmay Medhin

**Affiliations:** a Center for Innovative Drug Development and Therapeutic Trials for Africa (CDT- Africa), College of Health Sciences, Addis Ababa University, Ethiopia; b Department of Internal Medicine, School of Medicine, College of Health Sciences, Addis Ababa University, Ethiopia; c Aklilu Lemma Institute of Pathobiology, Addis Ababa University, Ethiopia

**Keywords:** INSTIs, Combination antiretroviral therapy, Type 2 diabetes, Dolutegravir, Raltegravir, Elvitegravir

## Abstract

**Objectives:**

The development of diabetes mellitus (DM) in patients taking integrase strand transfer inhibitors (INSTIs) has raised concerns. It's critical because, in most guidelines, INSTIs are the preferred third agent at first-line regimens. This study investigates the excess risk of developing DM among people living with HIV (PWH) on INSTIs-based regimens compared to those with other combination antiretroviral therapies (cART).

**Methods:**

A search from PubMed, clinicaltrials.gov, Latin America and Caribbean health sciences literature, Cochrane, and google scholar to retrieve case-control and cohort studies were done. The literature search was performed for studies from January 2007 to January 2021. Data were extracted from studies and pooled as risk ratios (RR) with a 95% confidence interval (CI) using Stata 14 software. The protocol was registered in PROSPERO, ID: CRD42021230282.

**Results:**

This review included ten studies, resulting in 62 400 participants. There was no significant difference in the incidence of DM between participants receiving INSTIs-based regimens versus other cARTs (RR 0.97, 95% CI: 0.92–1.03; participants = 50 958; studies = 4; I^2^ = 86.8%, chi-square = 22.67). There is no statistically significant difference in DM among people treated with INSTIs-based regimens compared to those treated with boosted protease inhibitors (PIs)-based regimens (RR 0.97, 95% CI 0.92–1.03; participants = 49 840; studies = 3; I^2^ = 89.3%, chi-square = 18.65). DM incidence was lower in INSTIs-based regimens than in those using non-nucleoside reverse transcriptase inhibitors (NNRTIs)-based regimens (RR 0.80, 95% CI 0.69–0.91; participants = 42 346; studies = 2; I^2^ = 0%, chi-square = 0.18).

**Conclusion:**

The present review shows a nonsignificant difference in the incidence of DM in patients receiving INSTIs-based regimens compared to other regimens. However, there was a lower incidence of DM in the INSTIs group compared to the NNRTIs-based and PIs compared to the NNRTIs-based. When the INSTIs drugs dolutegravir, raltegravir, and elvitegravir were compared, there was a lower incidence of DM in raltegravir compared with elvitegravir.

## 1 Background

HIV/AIDS has a devastating global impact on health. It has caused approximately 39 million deaths, and more than 36 million live with the virus globally [[1], [2], [3]]. The 20.7 million people living with HIV (PWH) in sub-Saharan Africa account for 67% of the global HIV prevalence. Yearly in this region alone, there are 730 000 new HIV infections and 300 000 AIDS-related deaths. Worldwide, 73% of adults have access to combination antiretroviral therapy (cART) [4].

There have been advances in cART and progress globally toward implementing treatment-as-prevention programs. Despite the above efforts, approximately 2 million people are newly infected with HIV every year globally [1,3,5]. Access to cART has increased survival for PWH [[6], [7], [8]]. With the longer life expectancy made possible with cART, many people living with HIV face an increased burden of noncommunicable diseases (NCDs) [6].

People with HIV are more likely to develop diabetes mellitus (DM) than the general population because of multiple factors, including HIV-1, lipodystrophy, heightened inflammation, increasing prevalence of obesity, hepatitis C co-infection, and racial/ethnic preference [9]. In the Multicenter AIDS Cohort Study, insulin resistance markers were higher in all groups of HIV-infected men than HIV-uninfected control subjects, even those not receiving cART, suggesting an effect of the viral infection [10]. In the ten-year diabetes incidence study, lipohypertrophy, lipoatrophy, and elevated BP were associated with DM [11]. A longitudinal observational cohort in Latin America found a high incidence of the following outcomes: impaired fasting glucose, DM, overweight, and obesity following cART initiation [12].

Insulin is a hormone that regulates blood sugar/glucose, resulting in raised blood sugar [13]. Globally, the total number of people living with DM has risen from 108 million in 1990 to 422 million in 2014, with the prevalence rising more in low- and middle-income counties than in high-income counties [14]. Furthermore, traditional metabolic disease risk factors intersect with HIV-specific risk factors in PWH, including metabolic perturbations related to cART [15,16]. Certain protease inhibitors, such as indinavir (IDV), lopinavir (LPV), and ritonavir (RTV), have been shown to reversibly induce insulin resistance, probably by inhibiting glucose translocation through GLUT4 [17]. Nucleoside reverse transcriptase inhibitors (NRTIs) such as zidovudine and stavudine directly and/or indirectly affect glucose metabolism [17]. Disruptions of glucose and body fat metabolism in PWH have been observed since the advent of cART. Older cART regimens contributed substantially to insulin resistance and body composition changes, and the current regimens have more subtle effects on glucose and fat metabolism [18].

INSTIs are now included in most cART combinations in both naive and experienced PWH. It is because of relatively high tolerability, a higher genetic barrier to resistance, and a greater likelihood of sustained treatment success than other classes [19,20].

The development of DM in INSTIs use is not well understood. The interaction between dolutegravir (DTG) and melanocortin four receptor (MC4R) in vitro and binding of radiolabeled α melanocyte-stimulating hormone (MSH) to MC4R may explain weight gain, which is, in turn, a risk factor for the development of DM [21]. On October 16, 2007, the US Food and Drug Administration (FDA) approved raltegravir (RAL) to treat HIV infection with other cART agents [22]. Since 2016, there has been a concerted effort to implement dolutegravir-based first-line cART regimens in low and middle-income countries following the WHO guidelines released in 2016 and 2018 [23]. South Africa and Uganda have amended their cART guidelines to transition to dolutegravir-based first-line regimens and away from efavirenz-containing regimens [23]. In the review, we aimed to synthesize the literature on the effects of INSTIs on insulin sensitivity and the onset and incidence of DM in patients with HIV. We compared these results with other cART drug classes.

## 2 Methods

### 2.1 Patient and public involvement

None.

Researchers followed the Preferred Reporting Items for Systematic Reviews and Meta-Analysis Protocols (PRISMA) Guideline [24,25]. The protocol for this systematic review and meta-analysis has been registered with the International Prospective Register of Systematic Reviews (PROSPERO) database, ID: CRD42021230282.

### 2.2 Eligibility criteria

Researchers included longitudinal cohort studies (prospective and retrospective) and case controls conducted globally and in all settings. Clinical trials were not included because there were no completed published articles. The studies recruited adult individuals who live with HIV on cART, with no restrictions on doses or regimens, and evaluated the incidence of DM in these individuals.

• **Participants:** All studies recruited PWH and cART. No restrictions on doses or regimens. The researchers excluded studies that recruited patients with comorbidities such as TB and other opportunistic infections, pregnancy, or breastfeeding patients. Those diagnosed with diabetes mellitus at baseline, who had been diagnosed with type 1 or juvenile diabetes mellitus, gestational diabetes, and pre-clinical studies.
• **Setting**: Global studies were included regardless of the continent and region. All studies were written in the English language.
• **Study design**: Cohorts and case-control studies were reviewed.
• **Publication dates** from January 2007 to January 2021.
• **Intervention/exposure:** The use of cART with standard doses of the drugs and regimens is inclusive. All cART contains two nucleoside reverse transcriptase inhibitors (NRTIs). So, Drug classes PI, NNRTI, and INSTIs were singled out as the third drug in the cART combination.
• **Outcome:** The following definitions and outcomes were used.

**Diabetes mellitus (DM)** – DM in the primary manuscripts was defined by evidence of glycosylated hemoglobin (HbA1c) ≥6.5%, initiation of diabetes-specific medication, or new DM diagnosis along with diabetes-related medication (to exclude prediabetes from the outcome) [26]. We also used fasting plasma glucose (FPG) 126 mg/dL or oral glucose tolerance test (OGTT) 200 mg/dL or random plasma glucose (RPG) ≥200 mg/dL in diagnosis. DM diagnoses were established using physician diagnosis mapped to the International Classification of Diseases, the 10th Revision codes [27], modified from the standard criteria for DM diagnosis from the American Diabetes Association [28] and expert Committee on the Diagnosis and Classification of Diabetes mellitus [29].

**Hyperglycemia** – To confirm new-onset hyperglycemia in the primary manuscripts, clinical charts of all patients with incident hyperglycemia were verified by two clinicians [30]. The diagnosis was defined as specific guidelines [31].

### 2.3 Primary and secondary objectives

The primary objectives were to evaluate the incidence of DM in HIV individuals on INSTIS and compare it with the other drug classes. The primary comparison was between INSTIs -based regimens and non-INSTIs-based regimens.

The secondary objectives were to evaluate the incidence of DM across the other cART classes. The comparison was specifically INSTIs versus PI and INSTIs versus NNRTI- based regimens. The other secondary objective was to evaluate the onset of hyperglycemia across cARTs.

### 2.4 Search strategy

A search from PubMed, clinical trials.gov, Latin America and Caribbean health sciences literature, and google scholar was done.

The search strategies in PubMed for the MeSH terms and text words were “diabetes [Text Word]' OR “diabetes mellitus” [MeSH Terms] OR (“Diabetes Mellitus, Type 2" [Mesh]) AND “integrase inhibitors” [MeSH Terms] OR HIV integrase inhibitors [Text Word] AND antiretroviral [Text Word] OR “Antiretroviral Agents” [Mesh] OR “Antiretroviral Therapy, Highly Active” [Mesh] AND “incidence” [Text Word] OR “Incidence” [Mesh] AND “Epidemiology” [Mesh] AND “epidemiology” [Subheading] AND “Cohort Studies” [Mesh] AND rate [All Fields] OR “Incidence” [Mesh].

### 2.5 Study selection, data collection, and data analysis

Data management and analysis were done with The Cochrane Handbook for Systematic Reviews of Interventions [32], Stata 14 and Mendeley. Two authors independently reviewed the results, and disagreements were resolved through discussion. When clarification was necessary, the corresponding authors of the manuscripts were contacted [33].

### 2.6 Data extraction and management

Data extraction and rating for the certainty of the evidence were performed by two authors independently by screening titles and abstracts of cohorts and case-control studies about HIV and diabetes to minimize the likelihood of error. Data extracted included participants, interventions, methods and outcomes, author and year of publication, country, study design, data collection, participants, follow-up duration, interventions, drugs, and treatment outcomes. Information was extracted using a structured data extraction format adapted from Cochrane. Disagreement between authors was resolved through discussion and consensus. For dichotomous outcomes, the number of occurrences of diabetes (event) and the total number of participants in the particular treatment group were documented.

### 2.7 Treatment of missing data

When the information sought from available reports about the study design and relevant data elements was missing, mail contacts with the investigators were made to request the data.

### 2.8 Assessment of risk of bias in included studies

Based on critical domains, the two authors independently judged these risks as low, unclear, or high [34]. The Cochrane Collaboration's tool for assessing the risk of bias in longitudinal studies was used. These included the following questions: was the selection of exposed and non‐exposed cohorts drawn from the same population? Can we be confident in the assessment of exposure? Can we be confident that the outcome of interest was not present at the start of the study? Did the study match exposed and unexposed for all variables associated with the outcome of interest, or did the statistical analysis adjust for these prognostic variables? Can we be confident in assessing the presence or absence of prognostic factors? Can we be confident in the assessment of the outcome? Was the follow-up of cohorts adequate? Were co‐Interventions similar between groups? [35] In all cases, an answer of 'Yes' will indicate a low risk of bias, and an answer of ‘No’ will indicate a high risk of bias. Studies were checked for evidence from multiple publications.

### 2.9 Measures of treatment effect

Hyperglycemia and diagnosis of diabetes were the primary outcomes of the review. RR was used to summarize the dichotomous outcomes. Results of the outcomes are presented as forest plots with summary statistical estimates and 95% conﬁdence intervals.

### 2.10 Assessment of heterogeneity

Heterogeneity was assessed by calculating Chi^2^ (threshold p > 0.1) and I^2^ statistics (threshold I^2^ > 40%), with values greater than 50% considered as substantial heterogeneity (I^2^ > 50%), it was identified and reported.

### 2.11 Data synthesis

A systematic narrative synthesis was provided in which summary results were presented using texts, tables, ﬁgures, and forest plots. Studies were identified with the first author and the year of publication. The Mantel– Haenszel statistical method and effect measure risk ratio were employed for data analysis, synthesis, and creation of forest plot).

Risk of bias summary: review authors' judgments about each risk of bias for the included studies.

1. Was the selection of exposed and non‐exposed cohorts drawn from the same population?
2. Can we be confident in the assessment of exposure?
3. Can we be confident that the outcome of interest was not present at the study start
4. Did the study match exposed and unexposed for all variables associated with the outcome of interest, or did the statistical analysis adjust for these prognostic variables?
5. Can we be confident in assessing the presence or absence of prognostic factors?
6. Can we be confident in the assessment of the outcome?
7. Was the follow-up of cohorts adequate?
8. Were co‐Interventions similar between groups?

## 3 Results

### 3.1 Search results, study characteristics, and risk of bias

**Fig. 1:**
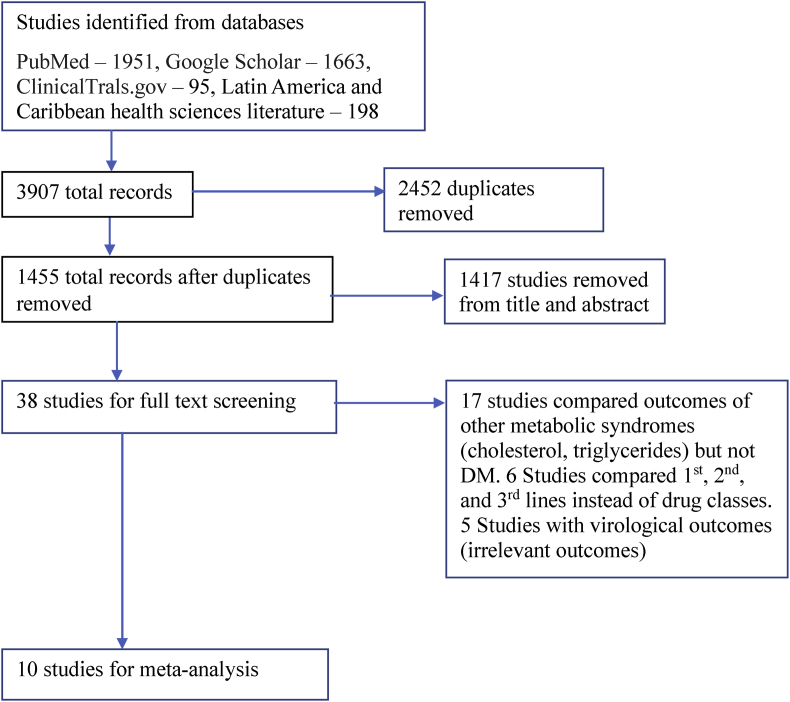
PRISMA flow diagram for article selection process.

A total of 3907 studies were identified from different databases, and 38 studies were full-text reviewed and assessed for eligibility. Ten studies that fulfilled the inclusion and exclusion criteria were part of the analysis (Fig. 1). The ten studies included had a total of 62 400 participants who were HIV positive on cART (Table 1). Table 2 summarizes our risks of bias. The quality of evidence was described as high, moderate, or low, depending on the heterogeneity.

**Table 1:** Summary characteristics of selected studies [30,31,[36], [37], [38], [39], [40], [41], [42], [43]].

Author and year of publication	Country	Study design	Year of data collection	Participants	Follow-up duration	Intervention drugs in the particular study	Treatment outcomes of DM or hyperglycemia (event of total)
Mohammed Lamorde et al., 2020 [30]	Uganda	Case-control	March 2018–March 2019	6647	One year	Dolutegravir non-dolutegravir first-line	16 of 3417
							1 of 3230
							**Hyperglycemia** total = 17
Ursenbach et al., 2020 [36]	France and overseas	Cohort	2009–2017	19 462	Eight years (median follow-up was 572days)	INSTIs	31 of 3403
						NNRTI	77 of 5601
						PI	157 of 10 458
							**DM** = 265 cases
Hsu et al., 2020 [37]	USA	Retrospective Cohort	August 1, 2013–March 31, 2018	7494	Five years (median follow-up was 1.5 years)	INSTIs	98 of 6527
						DTG	49 of 2816
						EVG/c	46 of 3504
						RAL	3 of 207
						bDRV (PI)	10 of 967
							**DM** = 108 cases
Rebeiro et al., 2020 [38]	USA and Canada	Cohort	January 2007–December 2017	22 884	Ten years (median follow-up was 1.6–3 years)	INSTIs	129 of 5184
						NNRTI	359 of 10 846
						PI	234 of 6855
							**DM** = 722 cases
					cohorts with >50 records	DTG	25 of 1210
					(INSTIS)	EVG/c	48 of 2315
						RAL	53 of 1081
							**DM** = 126 cases
Summers et al., 2020 [39]	USA	Retrospective cohort	2006–2017	1118	11 years (median follow-up of two years)	INSTIs	8 of 177
						non-INSTIs group	15 of 693
							**DM** = 23
Almeida SEM et al., 2009 [31]	Brazil	Retrospective cohort	January 2003 and March 2004	110	14 months (interquartile	NNRTI	17 of 84
					range 2–16 months)	PI	5 of 24
							**Hyperglycemia** total = 22
Tien et al., 2007 [40]	USA	Cohort	1994/5–2001/2	1524	Seven years	NNRTI	41 of 1420 (person time)
						PI	41 of 1641 (person time)
						NRTI	9
						No therapy	25
							**DM** = 116
Samad F et al., 2017 [41]	Canada	Retrospective cohort	1997–2015	703	18 years (median follow-up of 13 years)	NNRTI	30 of 202
						PI	86 of 460
						Others	16 of 41
							**DM** = 132
Bam NE et al., 2020 [42]	South Africa	Case-control	April 2015–March 2016	531	11 months	D4T/3TC/EFV	21 of 58
						AZT/3TC/EFV	40 of 58
						AZT/3TC/LPV	27 of 31
						AZT/3TC/RTV	19 of 23
						AZT/3TC/NVP	1 of 2
							69 of 359
							**DM** = 177
Han WM et al., 2019 [43]	Asia and Pacific region	Cohort	2003–2017	1927	14 years (follow-up of at least six months)	NRTI + NNRTI	102 of 1497
						NRTI + PI	22 of 364
						Others	3 of 66
							**DM** = 127

3 TC – lamivudine, AZT – zidovudine, bDRV – boosted darunavir, D4T – stavudine, DTG – dolutegravir, EFV – efavirenz, EVG – elvitegravir, LPV – lopinavir, NRTI – nucleotide reverse transcriptase inhibitors, NVP – nevirapine, RAL – raltegravir, RTV – ritonavir.

The definitions of the outcomes (DM and hyperglycemia) were according to the primary studies.

**Table 2:**
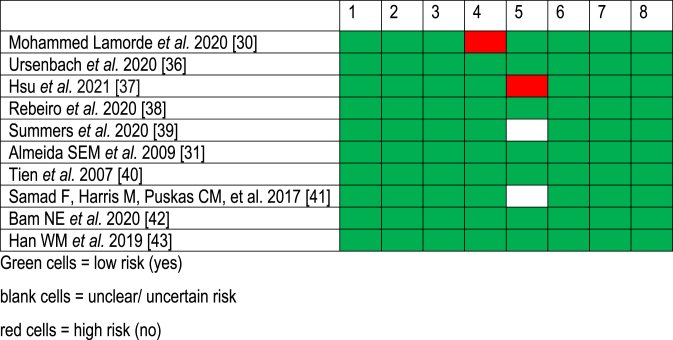
Risk of bias of the included studies [30,31,[36], [37], [38], [39], [40], [41], [42], [43]].

### 3.2 Effect of interventions

#### 3.2.1 Comparison of DM in INSTIs versus non-INSTIs cART

There was no overall significant difference between the two treatment groups in the four studies comparing incidences of DM in INSTIs and non-INSTIs cART [[36], [37], [38], [39]]. Studies by Ursenbach [36] and Rebeiro [38] show that DM cases were lower for participants treated with INSTIs, while Hsu [37] and Summers [39] were in favor of the other cART treatment group having a protective effect (reduced risk of DM). (RR 0.97, 95% CI 0.92–1.03; pcARTicipants = 50 958; studies = 4; I^2^ = 86.8%, chi-squared = 22.67). The heterogeneity with both chi^2^ (p > 0.1) and I^2^ (81–100%) was high.

#### 3.2.2 Comparison of DM in INSTIS versus PI

In three studies comparing incidences of DM in INSTIs and PI [ [[36], [37], [38]], there were no overall significant differences in participants treated with INSTIs or those treated with PI. Two studies [36,38] were in favor of INSTIs reducing the risk of DM, and one study [37] was for PIs reducing DM risk. (RR 0.97, 95% CI 0.92–1.03; participants = 49 840; studies = 3; I^2^ = 89.3%, chi-square = 18.65). The heterogeneity with both chi^2^ (p > 0.1) and I^2^ (81–100%) was high.

#### 3.2.3 Comparison of DM in INSTIS versus NNRTI

In the two studies comparing incidences of DM in INSTIs and NNRTI [36,38], results showed that there is a lower incidence of DM in the INSTIs group as opposed to the NNRTI group. Ursenbach [36] favors INSTIs to have the protective effect, and Rebeiro [38] favors NNRTI to have the protective effect. (RR 0.80, 95% CI 0.69–0.91; participants = 42 346; studies = 2; I^2^ = 0.00%, chi^2^ = 0.18). The heterogeneity was nonsignificant.

#### 3.2.4 Comparison of DM in PI versus NNRTI

**Fig. 2:**
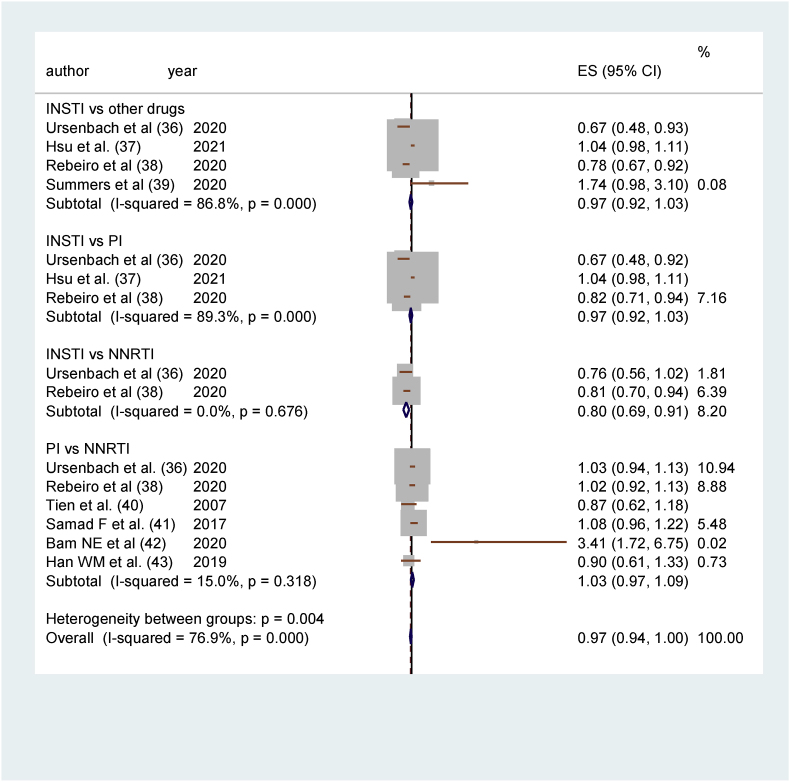
Forest plots showing comparison of DM in INSTI vs. non-INSTI ART [[36], [37], [38], [39]], INSTI vs. PI [[36], [37], [38]], INSTI vs. NNRTI [36,38], and PI vs. NNRTI [36,38,[40], [41], [42], [43]].

In the six studies comparing incidences of DM in PI and NNRTI [36,38,[40], [41], [42], [43]], incidences were generally lower for participants treated with PI than for those treated with NNRTI. Five studies favored PIs having a reduced risk of DM, and one study [42] favored NNRTIs to have a protective effect. (RR 1.03, 95% CI (0.97–1.09); studies = 6; I^2^ = 15.0%, chi-square = 5.88). Heterogeneity was nonsignificant (low) (Fig. 2).

#### 3.2.5 Comparison of DM in the INSTIs group

##### 3.2.5.1 Comparison of DM in DTG versus EVG

In the two studies comparing incidences of DM in DTG and EVG [37,38], there was no significant difference between the two treatment groups. Hsu [37] favors EVG and Rebeiro [38] favors DTG. (RR 1.11, 95% CI 0.92–1.29; studies = 2; I^2^ = 0.00%, chi-squared = 0.64). The heterogeneity was nonsignificant. (low).

##### 3.2.5.2 Comparison of DM in DTG vs. RAL

The two studies [37,38] showed no significant difference between the two treatment groups. The study by Hsu [37] favors raltegravir, while Rebeiro [38] favors DTG. (RR 0.97, 95% CI 0.90–1.03; studies = 2; I^2^ = 93.3%, chi^2^ = 14.89). The heterogeneity was high.

##### 3.2.5.3 Comparison of DM in RAL versus EVG

**Fig. 3:**
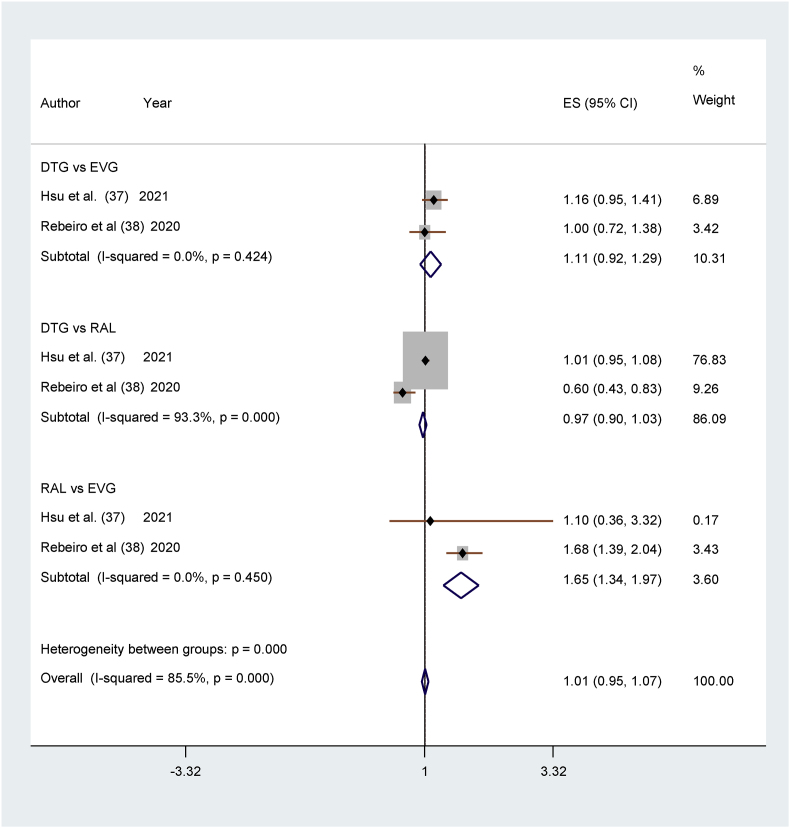
Forest plots showing Comparison of DM in INSTI group [37,38].

In the two studies comparing incidences of DM in RAL and EVG [37,38], there were lower incidences for participants treated with RAL than for those treated with EVG. (RR 1.65, 95% CI (1.34–1.97); studies = 2; I^2^ = 0.00%, chi-square = 0.67). The heterogeneity was nonsignificant. (low) (Fig. 3).

### 3.3 Hyperglycemia

Two studies, Mohammed Lamorde et al., 2020 [30] and Almeida SEM et al., 2009 [31], compared outcomes of hyperglycemia. Study [30] compared dolutegravir and non-dolutegravir-based regimens. The percentage of hyperglycemia in DTG was 0.46%, and non-DTG was 0.03%. In Almeida [31], the percentage of hyperglycemia in NNRTI was 20.2%, and PI was 20%.

## 4 Discussion

The review's focus was to determine the incidences of developing DM in PWH on the different cART available and compare the new class of INSTIs and the non-INSTIs group. The main findings of this meta-analysis are that the incidences of DM were without significant difference between the two treatment groups (INSTIs versus other cARTs). (moderate quality of evidence).

When comparing INSTIs and other groups, there was no statistical significance in incidences of DM in the participants (moderate quality of evidence). There was also no statistical significance when comparing INSTIs and PI. In contrast, incidences of DM were significantly lower in INSTIs compared to NNRTI (high-quality evidence). When comparing PIs with NNRTIs, the DM incidence was generally significantly lower for participants treated with PIs than those treated with NNRTIs (high-quality evidence). When comparing the individual drugs in the INSTIs group, dolutegravir vs. elvitegravir, incidences of DM were without significant difference (high quality of evidence). In dolutegravir vs. raltegravir, incidences of DM were without significant difference (moderate quality of evidence). In raltegravir vs. elvitegravir, incidences of DM were generally significantly lower for participants treated with raltegravir (high quality of evidence).

Heterogeneity in the four studies comparing DM in INSTIs vs. other drugs could be because, in two studies, there was no prior exposure to other medications (Ursenbach [36] and Rebeiro [38]). While in Hsu [37] and Summers [39], participants were previously exposed to other cARTs before the switch to INSTIs. In Summers [39], the study participants were only female.

Authors came across similar meta-analyses on incidences of DM, metabolic syndromes, and hyperglycemia that compared their findings among HIV and non-HIV patients. A study showed that the overall prevalence of metabolic syndromes among people living with HIV was 21.5% (95% CI 15.09–26.86) versus uninfected 12.0% (95% CI 5.00–21.00%), with substantial heterogeneity [44]. Two studies compared PIs and DM. One showed that PIs are associated with an increased risk of metabolic syndrome (MS), but no evidence of risk of DM increase was found. We know that metabolic syndromes usually happen before the development of DM and are a risk factor; studies with a longer follow-up duration may be needed to detect an association between PI use and the onset of DM [45]. Another was conducted in pregnant mothers, which revealed increased gestational diabetes (GDM) in studies using first-generation protease inhibitors (risk ratio 2.29, 95% CI: 1.46–3.58) and studies using the strictest diagnosis criteria, the National Diabetes Data Group criteria for 3-h oral glucose tolerance test (risk ratio 3.81, 95% CI: 2.18–6.67) [46]. O'Halloran et al. showed that INSTIs use was associated with an increased risk of new-onset diabetes mellitus/hyperglycemia in the six months following cART initiation [47].

This study focused on assessing any greater risks of developing DM in INSTIs. A recent case study [48] revealed a patient developed hyperglycemia three weeks after switching from efavirenz. Mohammed's findings [30] indicate more significant percentages of hyperglycemia in DTG, 0.46%, compared to non-DTG, 0.03%. Weight gain is one of the risk factors for the development of DM, and obesity is a health problem worldwide. INSTIs have been associated with weight gain. Supporting this is a study [15] showing that at 18 months, PWH on dolutegravir gained 6.0 Kg, compared to 2.6 Kgs for NNRTIs (*P* < 0.05) and 0.5 kg for elvitegravir (*P* < 0.05).

We also found similar case series on bictegravir [49]; The first case showed that four months after the switch, the patient presented to the emergency department (ED) with abdominal pain, blood glucose concentration was greater than 400 mg/dL, elevated blood ketone level was 4.5 mmoL/l. The second case showed that three weeks after the transition, he developed polyuria, polydipsia, and unintentional 15 Kg weight loss. Evaluations from the laboratory in the ED revealed hyperglycemia (>500 mg/dL) and elevated blood ketones (4.4 mmol/L). In the last case, two months after the transition, he presented to the ED for nausea, vomiting, polyuria, and polydipsia. His blood glucose concentration was >600 mg/dL, and the blood ketones were 4.2 mmol/L.

### 4.1 Study limitations

Most of the studies are longitudinal cohorts, retrospective cohorts, and case-control studies. There remains a need for randomized controlled trials, as some findings are heterogeneous. We also acknowledge that studies with INSTIs are limited, and there is a need to conduct more studies. When a subgroup analysis on the different drugs of INSTIs was done, the authors managed to get two studies for comparison. No study in this meta-analysis investigated the association between bictegravir and type 2 DM.

## 5 Conclusions

The present systematic review and meta-analysis show no significant difference in the incidences of DM in patients receiving INSTIs-containing cART regimens compared to other cART regimens. Among the INSTIs, there was also no significant difference between DTG and RAL or DTG and EVG. But there were lower incidences in RAL when compared to EVG.

## Credit author statement

Violet Dismas Kajogoo: Conceptualization, Investigation, Methodology, Software, Writing – original draft preparation. Wondwossen Amogne: Data curation, Supervision, Validation, Writing – review & editing. Girmay Medhin: Formal analysis, Supervision, Visualization, Writing – review & editing.

## Funding

This study was funded by the Center for Innovative Drug Development and Therapeutic Trials for Africa (CDT-Africa), 10.13039/501100007941Addis Ababa University, as a masters scholarship.

## Availability of data and material

Relevant data will be found in the manuscript and its supporting information files.

## Declaration of competing interest

The authors declare that they have no known competing financial interests or personal relationships that could have appeared to influence the work reported in this paper.
